# Political activity for physical activity: health advocacy for active transport

**DOI:** 10.1186/1479-5868-8-52

**Published:** 2011-05-29

**Authors:** Rosalina Richards, Linda Murdoch, Anthony I Reeder, Qa-t-a Amun

**Affiliations:** 1 Cancer Society Social and Behavioural Research in Cancer Unit, Department of Preventive and Social Medicine, Dunedin School of Medicine, University of Otago, PO Box 913, Dunedin, New Zealand

## Abstract

Effective health advocacy is a priority for efforts to increase population participation in physical activity. Local councils are an important audience for this advocacy. The aim of the current study was to describe features of advocacy for active transport via submissions to city council annual plans in New Zealand, and the impact of an information sheet to encourage the health sector to be involved in this process. Written submissions to city council's annual consultation process were requested for 16 city councils over the period of three years (2007/08, 2008/09, and 2009/10). Submissions were reviewed and categories of responses were created. An advocacy information sheet encouraging health sector participation and summarising some of the evidence-base related to physical activity, active transport and health was released just prior to the 2009/10 submission time. Over the period of the study, city councils received 47,392 submissions, 17% of which were related to active transport. Most submissions came from city residents, with a small proportion (2%) from the health sector. The largest category of submissions was in support of pedestrian and cycling infrastructure, design and maintenance of facilities and additional features to support use of these transport modes. Health arguments featured prominently in justifications for active transport initiatives, including concerns about injury risk, obesity, physical inactivity, personal safety and facilities for people with disabilities. There was evidence that the information sheet was utilised by some health sector submitters (12.5%), providing tentative support for initiatives of this nature. In conclusion, the study provides novel information about the current nature of health advocacy for active transport and informs future advocacy efforts about areas for emphasis, such as health benefits of active transport, and potential alliances with other sectors such as environmental sustainability, transport and urban planning and local communities.

## Introduction

Health advocacy is defined as a *'combination of individual and social actions designed to gain political commitment, social acceptance, and supportive policy and systems' *[1] and is a central component of successful health promotion [2].

Many factors underpinning the effective practice of advocacy remain relatively undocumented [3,4]. The 'chaotic reality' of advocacy involves a myriad of influences and opportunistic responses, which makes it an uneasy fit with traditional research methodologies [3,4]. Given the importance of advocacy for advancing health outcomes, however, the health sector urgently needs to build its capacity in this area.

One health issue where effective advocacy is a priority is for increasing population participation in physical activity [5]. Physical activity offers significant benefits for health and well-being across the lifespan [6-8]. The recently developed Toronto Charter for Physical Activity outlines a framework for action to advance the physical activity agenda [9]. The Charter is an advocacy tool to support physical activity initiatives and calls for action across four key areas; implementation of national policy and action plans, introduction of policies that support physical activity, reorientation of services and funding to prioritise physical activity and development of partnerships for action.

Active transport, which includes walking, cycling, skating, and self propelled wheelchairs, is an important subset of physical activity [10]. Active transport is of especial interest for encouraging sustained increases in participation, as it is a form of physical activity that can be built into everyday living. Active transport is associated with lower all cause mortality [11], increased fitness, decreased body weight and diastolic blood pressure among adults [12,13], and with greater physical activity among children [14]. Transport policies and systems that prioritise walking, cycling and public transport have been identified as one of the best investments for physical activity [15], and the health sector has an important role to play in supporting active transport initiatives [10,16,17].

Local governments are an important target for active transport advocacy [18]. Key functions of city councils in New Zealand include community well-being and development, roading and transport infrastructure and recreation and culture within their city [19]. As 86% of the New Zealand population reside in cities [20], the transport networks created by city councils are likely to play a significant role in supporting or impeding participation in active transport.

The focus of this paper is one avenue for advocacy to New Zealand city councils; the annual community consultation process. Early each year (March/April) city councils release a draft Annual Plan (outlining their planned activities and expenditures for the year ahead, midyear to midyear) or a Long Term Council Community Plan (LTCCP) (which plans for a ten year period) and request public submissions on the content of the plan. In addition to written submissions, it is also possible to speak in support of these to a meeting of the City Council representatives. Previous research has identified this submission process as an important opportunity for active transport advocacy [16]. The public nature of the submission process provides an opportunity to examine health sector involvement in advocacy to city councils. Furthermore, it provides an avenue to explore how the health sector may be mobilised to maximise their involvement.

Health advocacy is reliant on the availability of research evidence to support evidence-based advocacy [21,22]. Studies in science communication suggest that, for research evidence to inform policy and practice, information needs to be provided in a relevant and easily usable format [21]. Professional mobilisation of the health sector is likely to be encouraged by provision of information and facts to enable the physical activity workforce to advocate for policy changes, programmes and funding [4,5,23]. The Toronto Charter is one example of this, providing brief summaries of benefits for health, sustainable development and the economy [9]. Another example is web based resources provided by Active Living Research, including research syntheses, summaries and briefs for use in physical activity policy-making, practice and advocacy [24]. While these initiatives hold enormous potential, to our knowledge, there is little published evidence about the downstream impact of this type of information provision in supporting health advocacy.

**Figure 1:**
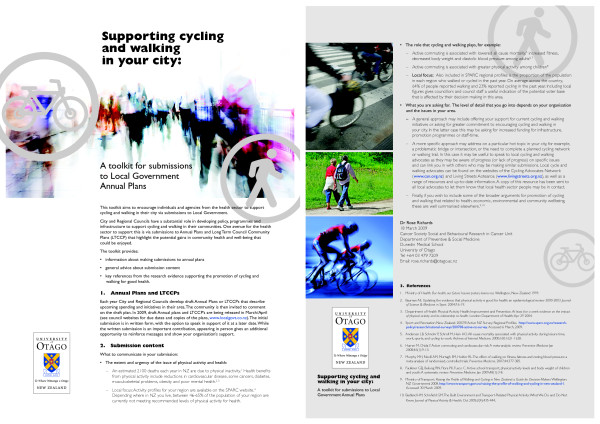
**Supporting cycling and walking in your city - A toolkit for submissions to Local Government Annual Plans**.

As part of the current study, an information sheet was developed that was specific to the city council submission process and health advocacy for active transport in New Zealand. The content of the information sheet encouraged participation in the submission process and summarised relevant international and national research evidence to support the case for active transport (Figure 1). The goal of the information sheet was to increase health sector participation in the submission process and encourage the use of evidence-based arguments for health and active transport.

The aim of the current study is to describe features of advocacy for active transport via submissions to city council annual plans in New Zealand for a three year period and the impact of an information sheet to encourage professional mobilisation of the health sector.

## Methods

### Participants

At the time of writing, there were 16 city councils in New Zealand; Auckland (population: 404,658), Manukau (population: 328,968), Waitakere (population: 186,444), North Shore (population: 205,605) Hamilton (population: 129,249), Tauranga (population: 103,632), Napier (population: 55,359), Palmerston North (population: 75,540), Porirua (population: 48,546), Upper Hutt (population: 38,415), Lower Hutt (population: 97,701), Wellington (population: 179,463), Nelson (population: 42,888), Christchurch (population: 348,435), Dunedin (population: 118,683), Invercargill (population: 50,328) [19,25].

### Methods

Submissions to annual plans and LTCCP's are public documents which can therefore be requested under the New Zealand Official Information Act (1982). For the purpose of this study, submissions to city councils were requested for annual plans or LTCCP's for the years 2007/08, 2008/09 and 2009/10. The request specifically asked for all submissions that were related to cycling and walking. Some councils chose to select, photocopy and send the requested submissions, others sent a submission summary index for the research team to identify the required submissions and others sent an electronic or paper copy of all the submissions received and the research team went through these for the relevant submissions. All submissions were then reviewed and categories of responses were created, for city, year, type of respondent, transport mode, what they were asking for and the reasons given for the request.

#### Information sheet

As part of this study an advocacy information sheet was developed and was released just prior to the 2009/10 submission time. The information sheet was specifically targeted towards individuals and agencies working in the health sector and included information about how to make a submission to city council, some of the evidence base related to physical activity, active transport and health, suggestions about sources for local data and issues to make the submission relevant to that city and contact details of local cycling and walking advocates in their city (Figure 1). The information sheet was distributed via several physical activity and health networks and organisations; Agencies for Nutrition Action, Obesity Action Coalition, Regional Sports Trusts, and Cancer Society of New Zealand, and Heart Foundation.

## Results

### Number of submissions to councils about active transport

A total of 2,784 submissions related to active transport were received by the 16 councils over the three year period (Table 1). This represents 17% of the total 47,392 submissions received on all topics. There was substantial variability between cities and years, in some cases representing more than half of submissions received, and in others being less than 1%.

**Table 1:** Number and Proportion of active transport related submissions for New Zealand cities over three years

	2007/08			2008/09			2009/19		
	walk/cycle	total	%	walk/cycle	total	%	walk/cycle	total	%
Auckland City	80	428	19	151	852	18	71	633	11
Hamilton City	17	201	8	30	193	16	46	384	12
Hutt City	106	1152	14	66	1262	5	107	938	11
Manukau City	6	364	2	3	1658	<1	15	19802	<1
Napier City	42	86	49	11	51	22	13	99	13
North Shore City	19	203	9	17	280	6	116	532	22
Palmerston North	16	150	11	35	445	8	65	544	12
Porirua City	31	51	61	41	57	72	278	492	57
Tauranga City	6	86	7	34	4078	1	70	1366	5
Upper Hutt City	9	103	9	6	44	14	11	107	10
Waitakere City	52	498	10	93	350	27	19	352	5
Wellington City	61	987	6	43	438	10	91	503	18
Christchurch City	7	161	4	18	541	3	148	1385	11
Dunedin City	124	800	16	131	531	25	160	812	20
Invercargill City	5	187	3	13	189	7	19	356	5
Nelson City	65	1111	6	53	261	20	164	1289	13

### Sources of submissions to councils about active transport

The majority of submissions identified were from private residents/households (78%, n = 2163), and community boards/groups (14%, n = 375), with smaller proportion coming from the health sector (2%, n = 61), business organisations (2%, n = 47), sport and recreation (Regional Sports Trusts/Sport and Recreation New Zealand) (1%, n = 32) and others (4%, n = 106). The majority of submissions related to active transport were in support of active transport initiatives, with only 5% in opposition (n = 150)

### Requests made in submissions to councils about active transport

Multiple requests were allowed for each submission, with 6,466 in total recorded. Of those in support of active transport (97%, n = 6263) the largest proportion were in support of a local cycle/walkway (18%, n = 1164), cycleway (20%, n = 1267), walkway/footpath (9%, n = 565) or were more generally in support of active transport (5%, n = 286)

Additional comments specifically related to cycling were about aspects of cycleway design (3%, n = 207) including surface coatings, lane width, drain covers/guttering, signage, bridge crossings and lighting. Maintenance was another issue (1%, n = 54) including sweeping glass and shingle off roads, enforcement of no-car parking in cycle lanes and vegetation trimming. Finally there were suggestions for additional facilities (7%, n = 424) including affordable bike hire/share schemes, bike parks and stands, lockers/showers, bike racks on buses/taxis/trains and Mountain bike/BMX/skateboard facilities.

Additional comments specific to walking included issues with the design of pedestrian facilities (3%, n = 200), such as street lighting, crossing design, pedestrian air-bridge/underpasses, and footpath surface material. There were also requests to 'pedestrianise' streets (n = 13%, n = 829). Maintenance of walking facilities was also an issue (5%, n = 302), including; clearing glass/litter and vegetation, dog control and minimising car parking and clutter on footpaths. Other facilities for pedestrians were also suggested (1%, n = 79) including streetscape beautification, seating, maps/brochures/signs, toilets and drinking fountains.

Other themes in responses included the need for funding for active transport (5%, n = 303), provision of safety education for drivers, cyclists and pedestrians (2%, n = 143), measures to reduce speed or bypass other traffic (3%, n = 180) and finally comment on the need for strategic planning, implementation plans and travel surveys to support ongoing improvements (2%, n = 103).

### Arguments in support for active transport

The reasons given for submission requests were also recorded. The most common reason given was health (38%, n = 1989), a substantial proportion of this (70%, n = 1384) was reduction in injury risk, with other aspects including reducing obesity, improving physical activity and improvements in personal safety and facilities for people with disabilities. Other categories of responses included environmental sustainability (6%, n = 329), accessibility (5%, n = 264), economic benefits (3%, n = 155), sport development (4%, n = 200), reducing travel costs/car use (5%, n = 247).

### Opposition to active transport

While the overwhelming majority of submissions regarding active transport were in support of it, and measures to encourage this, there were some that expressed at least some opposition (5%, n = 150). The majority of the 203 requests (89%, n = 180) were in opposition to provision of walking or cycling facilities (walkways//cycleways/crossings) for reasons of cost, perceptions of lack of use, and concerns about environmental damage or wildlife disturbance from track development. The second main point of opposition was to facilities being shared between cyclists and pedestrians (5%, n = 10), due to concerns about injury risk to pedestrians.

### Professional mobilisation of the health sector

A proportion of the submissions over the three year period were identified as being from the health sector (2%, n = 61). Respondents included Public Health Units, District Health Boards, health coalitions, disability advocacy organisations, health related charitable organisations, and health care providers. A total of 155 requests were made, most asking for council to support cycling and walking (39%, n = 60), support a cycleway or walkway (34%, n = 52). Other requests related to aspects of walkway design (14%, n = 22), walkway maintenance (7%, n = 11), implementation of active transport strategies (6%, n = 10) and prioritisation of cycling and walking over other modes of travel (5%, n = 8).

Arguments supporting requests submitted by the health sector included benefits for general health and well being (n = 34, 22%), reductions in injury risk (19%, n = 30), physical activity (14%, n = 22), obesity prevention (7%, n = 11), personal safety (3%, n = 5). Accessibility was also an issue, including accessibility to places and facilities (8%, n = 13), integrating networks (4%, n = 6), and social connectivity (3%, n = 5). A final category was to support environmental sustainability (10%, n = 15) and reduce air pollution (3%, n = 5).

### Impact of the Information Sheet

Of the 61 health submissions received 28% (n = 17) were in 2007/08, 33% (n = 20) in 2008/09 and 39% (n = 24) for the 2009/19 LTCCP. When the 2009/19 submissions, which were submitted after the circulation of the information sheet, were reviewed there were 3 submissions (12.5%) that had quoted research evidence summarised in the information sheet. In each case the sentences which outlined health benefits of active transport and referenced the relevant research articles were included verbatim; therefore it is unlikely that these were gained from another source. Of the three submissions, one had not submitted in the prior two years, and two had submitted previously, but had not included any health research evidence to support their arguments for physical activity or active transport.

## Discussion

The annual plan/LTCCP consultation process receives thousands of submissions each year, related to all aspects of life in the city; from parks and tourism to libraries and public toilets. Over the study period described here, one in every six submissions was related to cycling or walking, suggesting that active transport has become a high profile agenda item for many New Zealand cities, as it has in other countries [10].

Most submissions offered support for pedestrian and cycling infrastructure, design and maintenance of facilities and additional features that would further support use of these transport modes. These requests are consistent with other research studies which have highlighted the importance of built environment in facilitating participation in active transport [10,26-29]. Other categories of submissions echoed the Toronto charter's emphasis on the importance of supportive policy and service environments for active transport initiatives [9], highlighting issues of adequate funding, strategic and implementation plans, and initiatives to educate road-users about safety and courtesy to other transport modes.

Health arguments featured prominently in justifications for active transport initiatives. These included concerns about injury risk, obesity, physical inactivity, personal safety and facilities for people with disabilities. Previous research has identified a tendency for emphasis of injury risk over health benefits [16,30] and this was also true in the total sample in the current study. While not disputing the importance of reducing injury risk, it would appear that there is a need for greater emphasis of the health benefits of active transport. Inclusion of health effects in assessment of transport and urban design interventions has the potential to be a powerful advocacy tool [17], and is one which the health sector is ideally placed to support.

An important aspect of advancing the physical activity agenda is the development of partnerships to support change [9]. This study identified some important areas for coalition within the health sector, namely between physical activity, injury prevention, obesity, and disability advocates. The arguments for active transport documented here also support the potential for alignment with other agenda's such as environmental sustainability [31,32], urban and transportation planning [4,33,34], economics [4,9] and sports development [35]. Another important partner in the development of local policies and programmes is local communities [17]. It is encouraging to see that, in many New Zealand cities, there was strong representation from city residents who were sufficiently motivated to write to their city council in support of active transport initiatives.

A small proportion of responses were in opposition to active transport initiatives. Identification and understanding of opposition arguments is an important part of advocacy efforts, firstly to guide attempts to find win-win scenarios where the wishes of both parties are met, or alternatively to allow counterarguments to be developed to limit the impact of entrenched opposition [36]. Similar to previous studies, the cost or resources needed to carry out active transport initiatives was a concern [10,16]. In New Zealand, homeowners pay an annual fee to the local council to help pay for local services. Opposition arises when the cost of cycling/walking initiatives is perceived as contributing to ongoing rises in costs to householders. A second point of opposition was perceptions that facilities were not needed as few cyclists or pedestrians currently use a particular route. Latent demand can be a difficult concept to communicate, particularly when a facility may form part of a larger network and increases in active transport users might not be realised until the entire network is completed [16]. Final areas of concern were for conflict between cyclists and pedestrians and potential environmental damage or wildlife disturbance from track development. These issues are important to consider in facility design, particularly where multiple active transport modes and/or city green-space might be marginalised into relatively small areas of the urban space.

While health arguments are prominent, the health sector comprised a small proportion of submitters (2%). Submissions from local health practitioners and agencies are likely to be particularly influential because they are a respected part of their community and are seen as an independent voice [16]. This relatively low degree of involvement from the health sector, however, seems unlikely to make a substantial impact on city council activities, in particular, in those cities where prioritisation of motorised transport or a 'car culture' is strongly embedded as a default position [10,16]. The information sheet used here to facilitate participation in the submission process, did not appear to markedly increase participation, however, it was able to support the presentation of evidence-based health arguments for active transport among a proportion of the health sector submissions (12.5%). Given the relative simplicity and low profile nature of the information sheet distribution this is an encouraging finding and offers tentative support for the role of summarising research evidence in a user friendly manner to support professional mobilisation efforts.

There are several limitations to this study. First, a 'big picture' of submissions across New Zealand is described here, however, it is important to note that the contexts for advocacy differ markedly across the 16 cities studied. A complementary approach would be the development of case studies of different cities to capture more detailed view of advocacy, as has been done elsewhere [37]. While the current study describes features of current advocacy, it was not able to capture the downstream impact of this advocacy on city policy and practice; this may also be more appropriately examined in a case study approach. It is also recognised that the submission process observed here is only one avenue of influence for the health sector and there are several other activities of interest, for example media campaigns, involvement in advisory committees/forums and individual engagement with decision-makers. Another limitation is in variability in the selection of submissions, with some identified by councils and others by the research team. While the selection criteria was straightforward (all submissions relevant to cycling or walking), there was potential for some relevant submissions to have been excluded in council selected samples.

Finally, there is a need to continue to improve tools such as this information sheet to increase their effectiveness as advocacy tools. There are two aspects to these resources, informational and motivational. The informational aspect involves providing easy access to evidence-based arguments to support active transport. Health professionals are typically busy and unlikely to have time to accumulate and summarise the research evidence themselves. They are also likely to be accustomed to working in an evidence-based manner and may, if evidence is not readily available, be reluctant to advocate in their professional capacity. The information sheet in this study was relatively simple, listing a few key references, however, informational resources would ideally be developed using systematic processes for reviewing literature and synthesis of research across all studies [38]. The second important purpose of a resource is motivational, that is, to convince a health professional that active transport is relevant to their role in health and that their involvement in advocacy is appropriate and important. In addition to the content, the context of how the information is delivered is also important. If a resource is delivered via a respected professional organisation or there is a respected 'champion' within a profession encouraging peers to be involved, individuals may be more likely to participate. In the current study, the information sheet was delivered electronically via physical activity and nutrition networks. Other avenues of dissemination, such as publication in professional newsletters or via organisations such as the Royal New Zealand College of General Practitioners may have increased uptake. Future research would benefit from involving the health professionals who are being targeted by the information sheet, to find out about barriers to advocacy, what types of information is useful, and what are the best means for disseminating this information.

To conclude, this study was an in-depth exploration of this advocacy through one avenue to one audience. Many features of the study findings were in line with previous research, but obtained from the novel source of formal submissions to city councils. Health sector involvement in this process was described, as well as suggestions about how prominent this was amongst the volume and variety of submissions viewed by city council decision-makers. There is scope for increased participation in evidence based health advocacy, particularly in highlighting the potential health benefits from active transport. It is encouraging to document some impact from the information sheet developed here, and offer some early evidence for the efficacy of initiatives to synthesise research into a form relevant to advocates and policy makers. In advocating for active transport, health is not operating in isolation. Several opportunities for partnership are identified between health and other sectors; along with potential opposition, which must be acknowledged and addressed. Perhaps the most inspiring aspect of these study findings is that health advocacy for active transport is currently being led by city residents themselves. The challenge now is for the health sector to bring its full weight to bear in support of these efforts.

## Competing interests

The authors declare that they have no competing interests.

## Authors' contributions

RR conceived of the study, participated in design, data collection, data analysis and drafted the manuscript. AIR participated in the design of the study and helped in drafting the manuscript. QA participated in the design, coordination and data collection, LM analysed the data and helped draft the manuscript. All authors read and approved the final manuscript.
